# Correlation between Triclosan (TCS) exposure and endometriosis

**DOI:** 10.12669/pjms.39.6.7170

**Published:** 2023

**Authors:** Kailai Ma, Yan Cheng, Hongling Guo, Jianguo Wang, Tingting Yu

**Affiliations:** 1 Kailai Ma, Department of Gynaecology, Taicang First People’s Hospital, Suzhou 215400, Jiangsu, China; 2 Yan Cheng, Department of Gynaecology, Taicang First People’s Hospital, Suzhou 215400, Jiangsu, China; 3 Hongling Guo, Department of Gynaecology, Taicang First People’s Hospital, Suzhou 215400, Jiangsu, China; 4 Jianguo Wang, Department of Gynaecology, Taicang First People’s Hospital, Suzhou 215400, Jiangsu, China; 5 Tingting Yu, Department of Gynaecology, Taicang First People’s Hospital, Suzhou 215400, Jiangsu, China

**Keywords:** Endometriosis, Triclosan (TCS), Income

## Abstract

**Objective:**

To study the correlation of Triclosan (TCS) exposure with typing and staging of endometriosis, and with other potential influencing factors.

**Methods:**

This was a retrospective study. Thirty two patients that were diagnosed with endometriosis by laparoscopy or surgery in Taicang First People’s Hospital from May 2020 to December 2021 were enrolled in the endometriosis group, and patients who were confirmed free of endometriosis by surgeries for other purposes during the same period were enrolled as the control group. All blood samples were tested twice in two different vials. The association of TCS exposure level with occurrence, staging, typing of endometriosis, and income of the patients were analyzed.

**Results:**

Patients with endometriosis had significantly higher TCS exposure levels than the control group. TCS exposure level in patients with endometriosis was positively correlated with patient income), and was significantly higher in patients with Stage-IV endometriosis than in those with Stage-III and II diseases. TCS exposure levels showed no significant difference among patients with ovarian type, ovarian + peritoneal type, and deep nodular type endometriosis TCS exposure level in patients with endometriosis was positively correlated with the staging of the disease. TCS exposure was highly positively correlated with the staging of the disease in patients with ovarian type endometriosis and in patients with deep nodular endometriosis, but there’s no such correlation in patients with ovarian + peritoneal type endometriosis.

**Conclusion:**

TCS exposure level in endometriosis patients was higher than that in normal women, and is positively correlated with endometriosis staging and income of the patients.

## INTRODUCTION

Triclosan (TCS), also known as 2,4,4’-trichloro-2’-hydroxydiphenyl ether, has been widely used in recent years in the production of toiletries, daily necessities, medical equipment, packaging materials, etc.^1^, so human exposure to TCS has greatly increased. As a typical endocrine disruptor, TCS is also very stable in the human body, leading to a greater toxic effect on the human reproductive endocrine system.^2^ At present, TCS has been widely detected in human body fluids, suggesting exposure to environmental TCS pollution.^3^ In recent years, the incidence of endometriosis showed a significant growth trend, and the etiology and pathogenesis of endometriosis are still under study.^4^,^5^ Some studies have shown that environmental endocrine disruptors play a role in the occurrence of endometriosis.^6^

Endometriosis is a common gynecological disease in women of childbearing age, and is correlated with up to 35% of habitual dysmenorrhea or infertility cases.^7^,^8^ In recent years, the incidence of endometriosis showed a significant increasing trend, and has become a major cause of hysterectomy in women of childbearing age, seriously affecting the quality of life of the patients and posing a threat to human reproduction.^9^,^10^ Endometriosis is an estrogen-dependent disease, and it is currently believed that genetics, immune regulation and environmental pollutants all play a role in its occurrence. Previous studies have shown that environmental endocrine disruptors (EEDs) such as dioxin, poly Chlorinated biphenyls, bisphenol A, and phthalates can all increase the risk of endometriosis.^11^ Considering the similarities in structure and biological effects of TCS with traditional EEDS mentioned above^12^ and the current environmental TCS exposure level^13^, it is necessary to investigate the relationship between TCS and endometriosis, which is of great significance for the prevention and treatment of endometriosis. In this study, the correlation of environmental endocrine disruptors with the occurrence, staging, and typing of endometriosis has been studied.

## METHODS

This was a retrospective study. Patients with endometriosis diagnosed by laparoscopy or surgery in Taicang First People’s Hospital from May 2020 to December 2021 were selected. Patient data including demographic data, main clinical features were retrieved from electronic medical record systems. A total of 55 patients were enrolled in this study, including 32 females in the endometriosis group, and 28 female patients in the control group. The two groups were comparable in age (38.25±7.65 years vs. 36.85±7.09 years, p>0.05).

### Ethical Approval

The study was approved by the Institutional Ethics Committee of Taicang First People’s Hospital (No.: KY-2016-013; date: October 20, 2019), and written informed consent was obtained from all participants. Thirty two patients that were diagnosed with endometriosis by laparoscopy or surgery in the endometriosis group.

The control group included female patients who were confirmed to have no endometriosis by laparoscopy or surgery for other reasons in the same hospitals during the same period.

### Inclusion criteria:

- >18 years old and had regular menstrual cycle.
- Intact uterus (endometrial polyps and cervical are allowed).
- Has at least one intact ovarian.

### Exclusion criteria:

- Previously diagnosed with endometriosis.
- Breastfeeding.
- Had received hormone therapy in the past three months, and had no menstrual cramps after discontinuation.
- Obvious liver or kidney insufficiency, or suffering from malignant tumors, breast cancer, uterine fibroids, or gynecological diseases related to the imbalance of sex hormone secretion.
- Occupational exposure to TCS.

### Determination of TCS Exposure Levels

With informed consent, blood samples of the subjects were collected for measurement of TCS content, which reflects the TCS exposure level of the patients. Ten mililiter of anticoagulated blood and 10 mL of non-anticoagulated blood were collected for each patient and immediately sent to biological sample bank for aliquoting, the plasma into six tubes and the serum into five tubes. These biological samples were analyzed with HPLC-MS/MS by the biological laboratory of Shanghai Baoteng Biological Co., Ltd., using reagents like fluorescently labeled anti-CD4, CD25, FoxP3, IL-17A monoclonal antibodies, and AhR activator. All samples were divided into two batches to repeat the test, and the average value of the two tests was taken as the value of the patient’s TCS exposure level. All the procedures in both groups were performed by the same group of doctors. The maximum follow-up time for patients in both groups was six months. And case data collection ceased in June 2022.

### Statistical analysis

SPSS 21.0 statistical software was used for data analysis. Measurement data were represented as (*χ̅*±*S*), independent t-test or one-way analysis of variance was used for comparison between groups (LSD method was used for pairwise comparison); the correlation between two data sets was analyzed by Pearson correlation analysis or Spearman correlation analysis, with 0.3<|r|<0.5 being considered weakly correlated, 0.5<|r|<0.8 moderately correlated, and |r|>0.8 strongly correlated. P<0.05 was considered statistically significant.

## RESULTS

The TCS exposure level of the 32 patients with endometriosis was significantly higher than that of the 28 controls, showed significant difference (t=2.712, 0.009). TCS exposure level of patients with endometriosis was significantly positively correlated with their income (Pearson’s correlation coefficient r= 0.647, p<0.001). More specifically, TCS exposure of patients with ovarian type endometriosis was highly positively correlated with their income (Pearson correlation coefficient r= 0.776, p<0.001). Table I and Table II.

**Table-I:** Comparison of TCS exposure levels between the two groups of patients.

Groups	Number of patients	TCS exposure level (ug/L)
Endometriosis	32	0.69±0.34
Normal control	28	0.51±0.15
*t*		2.712
*P*		0.009

**Table-II:** Correlation between TCS exposure level and income of patients with endometriosis.

Type of endometriosis	Number of patients	Average income (10,000/year)	r	P
endometriosis	32	12.81±8.16	0.647	0.647
Ovarian type	17	12.76±9.46	0.776	<0.001
Ovarian + peritoneal type	8	12.14±5.50	0.447	0.267
deep nodular	7	13.69±8.28	0.366	0.420
Stage-II	8	7.36±3.21	0.088	0.835
Stage-III	14	12.10±7.65	0.174	0.551
Stage-IV	10	18.15±8.83	0.782	0.008

One-way ANOVA showed a significant difference among the three groups (F=5.809, p=0.008), and LSD pairwise comparison showed significantly higher TCS exposure levels in patients with Stage-IV endometriosis than in Stage-III and II patients (p=0.031, 0.012. Table-III.

**Table-III:** Correlation between TCS exposure level and staging of endometriosis

Groups	Number of patients	TCS exposure level (ug/L)
Stage-II	8	0.50±0.07
Stage-III	14	0.61±0.19
Stage-IV	10	0.95±0.47
*F*		5.809
*P*		0.008

***Note:*** LSD pairwise comparison showed significantly higher TCS exposure levels in Stage-IV than in Stage-III and II (p=0.031, 0.012).

One-way ANOVA showed no significant difference among the three groups (F=0.267, p=0.768).Table-IV. TCS exposure level was significantly correlated with the staging of endometriosis (Spearman’s correlation coefficient r was 0.596, p<0.001).

**Table-IV:** TCS exposure levels of the 3 types of endometrioses

Groups	Number of patients	TCS exposure level (ug/L)
Ovarian type	17	0.66±0.37
Ovarian + peritoneal type	8	0.67±0.29
deep nodular	7	0.77±0.34
*F*		0.267
*P*		0.768

More specifically, TCS exposure was highly positively correlated with the staging of the disease in patients with ovarian type endometriosis (Spearman’s correlation coefficient r=0.863, p<0.001) and in patients with deep nodular endometriosis (Spearman’s correlation coefficient r=0.839, p=0.018), but there’s no such correlation in patients with ovarian + peritoneal type endometriosis (p=0.819).Table-V.

**Table-V:** Correlation between TCS exposure level and endometriosis staging in patients with specific types of disease.

Type of endometriosis	Number of patients	TCS exposure level (ug/L)	r	P
endometriosis	32	0.69±0.34	0.596	<0.001
Ovarian type	17	0.66±0.37	0.863	<0.001
Ovarian + peritoneal type	8	0.67±0.29	-0.097	0.267
deep nodular	7	0.77±0.34	0.839	0.018

## DISCUSSION

By comparing the TCS exposure levels of patients with different stages of endometriosis, it was found that patients with Stage-IV had the highest level of TCS exposure, significantly higher than those with Stage-III and II disease. Actually, the average TCS exposure level (0.50 ug/L) in patients with Stage-II endometriosis was not so different from that of normal control (0.51 ug/L). These results suggest that severe (Stage-IV) endometriosis correlated with high TCS exposure levels, even among patients with endometriosis. TCS exposure levels showed no significant difference among patients with different types of endometrioses (ovarian, ovarian + peritoneal, and deep nodular).

This study demonstrated a positive correlation of TCS exposure with the staging of endometriosis, more specifically, TCS exposure was highly positively correlated with the staging of the disease in patients with ovarian type endometriosis and in patients with deep nodular endometriosis, but there’s no significant correlation between TCS exposure and disease staging in patients with ovarian + peritoneal type endometriosis, possibly because of limited sample size. The occurrence and development of endometriosis are closely related to the imbalance of steroid hormone secretion and regulation. TCS has endocrine disrupting effects, which may cause changes in the synthesis of human endometrial hormones and ovarian steroid hormones, and overproduction of estrogen in ectopic endometrial tissue.^14^

TCS exposure level was 35.3% higher in patients with endometriosis than in normal people (0.69 vs. 0.51 on average). And we believe that the increase in blood TCS level in patients with endometriosis is not accidental, but suggest higher TCS exposure in life and thus high TCS accumulation in the body. TCS is an EED that interferes with endogenous hormones, and is difficult to be degraded in the human body.^15^ In recent years, TCS has been considered one of the major causes of some endocrine diseases (including endometriosis). TCS exposure level of patients with endometriosis was positively correlated with their income, and it was speculated that the high-income group is more likely to be exposed to a large number of toiletries and daily necessities that contain TCS and the like. China is a major producer and a major consumer of TCS.^16^ Products containing TCS enter the surrounding environment after being used, and TCS may accumulate through the food chain, so when people eat or use such animal and plant products, they would be exposed to the threat of TCS.^17^ With the excessive use of EEDs in production and daily life and the continuous emergence of new EEDs, their potential effects on the human body (including the reproductive endocrine system) have attracted more attention from researchers.^18^

Animal experiments have demonstrated the disruptive effect of TCS on the reproductive endocrine system, as TCS exposure affected estrogen-mediated responses in adolescent female rats, resulting in increased uterine weight and corresponding histological changes (such as earlier vaginal opening).^19^ In female rats, TCS can enhance the effect of ethinyl estradiol to promote uterus growth, suggesting that TCS has estrogenic effects.^20^ Meanwhile, studies have also shown that TCS exposure has a significant effect on maternal-fetal interface trophoblast cells^21^, regulating the function of human chorionic trophoblast cells HTR8-SVneo^22^, and expression of 11β-HSD2 and HLA-G in human early pregnancy trophoblast cells.^23^ These studies suggested that TCS not only has estrogenic activity by directly interacting with receptors^24^, but also affects hormone synthesis and metabolism by interfering with the hypothalamic-pituitary-gonadal axis.^25^ Based on relevant studies of others and the results of this clinical study, it can be argued that TCS can induce abnormal estrogen metabolism in various ways, leading to estrogen-dependent growth and expansion of ectopic foci, which eventually develop into endometriosis.^26^

### Limitations

We only analyzed and discussed the cases admitted in our hospital. In the future clinical work, the sample size and follow-up period will be further increased and the effect of different therapies on the long-term effect and survival of patients further improved, so as to evaluate the benefits of this regimen to patients in a more comprehensive manner.

## CONCLUSION

In conclusion, TCS exposure level is higher in patients with endometriosis than in normal women, and is positively correlated with patients’ income and disease staging. In follow-up studies, a larger sample size is needed to further confirm the association between endometriosis and TCS exposure.

### Authors’ Contributions:

**KM and YC** carried out the studies, data collection, drafted the manuscript, and are responsible and accountable for the accuracy and integrity of the work.

**HG and** **JW** performed the statistical analysis and participated in its design.

**TY:** Data analysis, interpretation of data and draft the manuscript. All authors read and approved the final manuscript.

## References

[1] Russell AD (2004). Whither triclosan?. J Antimicrob Chemother.

[2] Weatherly LM, Gosse JA (2017). Triclosan exposure, transformation, and human health effects. J Toxicol Environ Health B Crit Rev.

[3] Zheng X, Yan Z, Liu P, Fan J, Wang S, Wang P (2019). Research Progress on Toxic Effects and Water Quality Criteria of Triclosan. Bull Environ Contam Toxicol.

[4] Mehedintu C, Plotogea MN, Ionescu S, Antonovici M (2014). Endometriosis still a challenge. J Med Life.

[5] Cheng F, Lu L, Wang H, Cheng H, Zhang D (2019). Expression and Significance of miR-126 and miR-145 in Infertility due to Endometriosis. J Coll Physicians Surg Pak.

[6] Chiang C, Mahalingam S, Flaws JA (2017). Environmental Contaminants Affecting Fertility and Somatic Health. Semin Reprod Med.

[7] Czyzyk A, Podfigurna A, Szeliga A, Meczekalski B (2017). Update on endometriosis pathogenesis. Minerva Ginecol.

[8] Siddiqui QUA, Anjum S, Zahra F, Yousuf SM (2019). Ovarian reserve parameters and response to controlled ovarian stimulation in infertile patients. Pak J Med Sci.

[9] Chapron C, Marcellin L, Borghese B, Santulli P (2019). Rethinking mechanisms, diagnosis and management of endometriosis. Nat Rev Endocrinol.

[10] Sakar MN, Oglak SC (2020). Letrozole is superior to clomiphene citrate in ovulation induction in patients with polycystic ovary syndrome. Pak J Med Sci.

[11] Wei L, Wu Q (2015). Reproductive toxicity of triclosan. Chin J of Family Plan.

[12] Alfhili MA, Lee MH (2019). Triclosan:An Update on Biochemical and Molecular Mechanisms. Oxid Med Cell Longev.

[13] Guerra P, Teslic S, Shah A, Albert A, Gewurtz SB, Smyth SA (2019). Occurrence and removal of triclosan in Canadian wastewater systems. Environ Sci Pollut Res Int.

[14] Forte M, Mita L, Cobellis L, Merafina V, Specchio R, Rossi S (2016). Triclosan and bisphenol a affect decidualization of human endometrial stromal cells. Mol Cell Endocrinol.

[15] Dann AB, Hontela A (2011). Triclosan:environmental exposure, toxicity and mechanisms of action. J Appl Toxicol.

[16] Yu Y, Huang Q, Wang Z, Zhang K, Tang C, Cui J (2011). Occurrence and behavior of pharmaceuticals, steroid hormones, and endocrine-disrupting personal care products in wastewater and the recipient river water of the Pearl River Delta, South China. J Environ Monit.

[17] Dhillon GS, Kaur S, Pulicharla R, Brar SK, Cledón M, Verma M (2015). Triclosan:current status, occurrence, environmental risks and bioaccumulation potential. Int J Environ Res Public Health.

[18] Dix-Cooper L, Kosatsky T (2019). Use of antibacterial toothpaste is associated with higher urinary triclosan concentrations in Asian immigrant women living in Vancouver, Canada. Sci Total Environ.

[19] Bedoux G, Roig B, Thomas O, Dupont V, Le Bot B (2012). Occurrence and toxicity of antimicrobial triclosan and by-products in the environment. Environ Sci Pollut Res Int.

[20] Li H, Li D, Huang XM, Guo D, Ni F (2018). Comparative study on hepatotoxicity and reproductive toxicity in the female rats exposed to triclosan during weaning to sexual maturity. Straits J Pre Med.

[21] Jiao T (2016). Effects of triclosan on trophoblast invasion at the maternal-fetal interface and its molecular mechanism. Nanjing Med Univer.

[22] Lin ML, Hua R, Zhou Y, Na J, Cai H, Quan S (2020). The effect of triclosan on the function of human chorionic trophoblast cells HTR8-SVneo. Chin J Prac Gyne and Obste.

[23] Yang Q, Wang Y (2016). Effects of triclosan on expression of 11β-HSD2 and HLA-G in human first trimester trophoblasts. J Shanghai Jiaotong Uni (Med Edi).

[24] Rolla E (2019). Endometriosis:advances and controversies in classification, pathogenesis, diagnosis, and treatment. F1000Res.

[25] Dai Y, Li X, Shi J, Leng J (2018). A review of the risk factors, genetics and treatment of endometriosis in Chinese women:a comparative update. Reprod Health.

[26] Zhu W, Zhou W, Huo X, Zhao S, Gan Y, Wang B (2019). Triclosan and Female Reproductive Health:A Preconceptional Cohort Study. Epidemiol.

